# Reply to: Therapeutic hypothermia after cardiac arrest: outcome predictors

**DOI:** 10.5935/0103-507X.20160039

**Published:** 2016

**Authors:** Rodrigo Nazário Leão, Paulo Ávila, Raquel Cavaco, Nuno Germano, Luís Bento

**Affiliations:** 1 Unidade de Urgência Médica, Centro Hospitalar de Lisboa Central - EPE - Lisboa, Portugal.

We would like to thank the interest paid to our study and the valuable comments regarding it.^(1)^ Herein, we clarify the comments and questions raised.

Despite advances in cardiopulmonary resuscitation, cardiac arrest is still associated with high morbidity and mortality.^(2)^ Survival of these patients depends on the quality of care, and despite the fact that basic and advanced life support have been the subject of intensive research, the main focus is currently on care provided post-recovery of spontaneous circulation.^(3)^ Therapeutic hypothermia has been shown to be effective in the prevention and reversal of neurological injury, cardiac protection, and mortality reduction.^(4)^ It has been recommended since 2003 in comatose outpatients with ventricular fibrillation. Subsequent studies have shown the benefits of its immediate use after spontaneous circulation is restored and with other initial rhythms, which led to the initiation of cooling in pre-hospital settings and in patients with other rhythms.^(5,6)^ This fact introduced a new variable in the so important prognosis evaluation of patients; thereby, we conducted a study to determine the validity of several markers that could be used to identify patients with poor prognosis who underwent therapeutic hypothermia after cardiac arrest. Thus, we studied the influences of the setting (in-hospital and out-of-hospital), time, rhythms, clinical evaluation, and biochemical, neurophysiological or imaging parameters on the final prognosis, using a population that was appropriate for these analyses.

In regard to the discussion, we agree with the observation that the initial temperature was not presented. We chose not to include the initial temperatures in the study because this parameter was not collected in a systematic manner, which could then bias the results. Nevertheless, we found that the initial temperatures of the patients ranged from 35.5ºC to 36.8ºC. Similarly to Perman et al., we observed that patients who reached the target temperature faster had worse prognosis.^(7)^ We hypothesized that this was due to the existence of more extensive and irreversible neurological damage, which would make the patient less reactive to temperature decrease, with fewer tremors and reduced need for sedation, thus allowing faster cooling.^(8)^ In fact, this hypothesis implies that the lower reactivity to temperature decrease may be a secondary prognostic factor for more severe neurological injury and, consequently, may determine a shorter time for the target temperature to be reached, consistent with the results of our study, with all the variables interconnected.^(7-10)^

As already mentioned, some studies suggest that the early onset of therapeutic hypothermia after cardiac arrest is a safe and beneficial treatment to reduce mortality and improve neurological outcomes, and this has led to its establishment in the pre-hospital setting. In our study, there was no improvement of the prognosis when the protocol was subjected to pre-hospital initiation, which is consistent with the results of several investigations.^(9-13)^ As for the study by Kim et al., the authors concluded that induction of pre-hospital hypothermia increased the time that the patient spent in the pre-hospital setting, the number of re-arrests that occurred during transport, and the occurrence of acute pulmonary edema, in addition to possibly delaying the implementation of interventions such as cardiac catheterization.^(11)^ In our study, pre-hospital induction of hypothermia did not delayed patient admission to our unit nor the implementation of coronarography, which was performed within 12 hours, as recommended by the European Society of Cardiology.^(14)^ Moreover, there were no cardiac arrests during transport or episodes of acute pulmonary edema. We also clarified that no patients included in the study experienced therapeutic limitation or withdrawal of support.

Recent studies, such as that by Nielsen et al., suggest that avoiding hyperthermia can be as beneficial as hypothermia.^(15)^ These data led to changes in the recommendations of the American Heart Association and the European Resuscitation Council, which then suggested a more liberal approach, allowing temperatures between 32 - 36ºC, according to the patient's case.^(16)^ We believe that the fact that we did not compare the temperature level with mortality and neurological outcomes represents a limitation that was not previously considered as the study was designed and initiated prior to the publication of the study by Nielsen et al. accordingly with the existing recommendations at the time.

Rodrigo Nazário Leão, Paulo Ávila, Raquel Cavaco, Nuno Germano and Luís Bento

Unidade de Urgência Médica, Centro Hospitalar de Lisboa Central - EPE - Lisboa, Portugal.
